# Metabolic Characterization of Intact Cells Reveals Intracellular Amyloid Beta but Not Its Precursor Protein to Reduce Mitochondrial Respiration

**DOI:** 10.1371/journal.pone.0168157

**Published:** 2016-12-22

**Authors:** Patrick M. Schaefer, Bjoern von Einem, Paul Walther, Enrico Calzia, Christine A. F. von Arnim

**Affiliations:** 1 Institute of Neurology, Ulm University, Ulm, Germany; 2 Central Facility for Electron Microscopy, Ulm University, Ulm, Germany; 3 Institut für Anästhesiologische Pathophysiologie und Verfahrensentwicklung, Universitätsklinikum Ulm, Ulm, Germany; Heinrich-Heine-Universitat Dusseldorf, GERMANY

## Abstract

One hallmark of Alzheimer´s disease are senile plaques consisting of amyloid beta (Aβ), which derives from the processing of the amyloid precursor protein (APP). Mitochondrial dysfunction has been linked to the pathogenesis of Alzheimer´s disease and both Aβ and APP have been reported to affect mitochondrial function in isolated systems. However, in intact cells, considering a physiological localization of APP and Aβ, it is pending what triggers the mitochondrial defect. Thus, the aim of this study was to dissect the impact of APP versus Aβ in inducing mitochondrial alterations with respect to their subcellular localization. We performed an overexpression of APP or beta-site amyloid precursor protein cleaving enzyme 1 (BACE1), increasing APP and Aβ levels or Aβ alone, respectively. Conducting a comprehensive metabolic characterization we demonstrate that only APP overexpression reduced mitochondrial respiration, despite lower extracellular Aβ levels compared to BACE overexpression. Surprisingly, this could be rescued by a gamma secretase inhibitor, oppositionally indicating an Aβ-mediated mitochondrial toxicity. Analyzing Aβ localization revealed that intracellular levels of Aβ and an increased spatial association of APP/Aβ with mitochondria are associated with reduced mitochondrial respiration. Thus, our data provide marked evidence for a prominent role of intracellular Aβ accumulation in Alzheimer´s disease associated mitochondrial dysfunction. Thereby it highlights the importance of the localization of APP processing and intracellular transport as a decisive factor for mitochondrial function, linking two prominent hallmarks of neurodegenerative diseases.

## Introduction

Alzheimer´s disease (AD) is the most frequent neurodegenerative disease and is characterized by a loss of memory function and learning ability. The two major histopathological hallmarks are neurofibrillary tangles consisting of Tau and senile plaques comprised of amyloid beta (Aβ) derived from the amyloid precursor protein (APP). APP is sequentially cleaved by beta-site amyloid precursor protein cleaving enzyme 1 (BACE1) and gamma secretase. The underlying pathogenesis is still not well understood and seems to be complex ranging from disturbances in cellular protein transport and clearance to cellular energy production. Focusing more on the energy metabolism it has been shown that the severity of disease is associated with progressive alterations in brain metabolism [1] of AD patients. These alterations comprise manifold cellular and molecular changes [2] for example glucose hypometabolism [3–5] and a reduction in the gene expression [6], protein level and activity [7, 8] of mitochondrial proteins. Taking into account the abundance of metabolic disturbances as early as in mild cognitive impairment [9] and the detrimental effects of the resulting energy imbalance on neuronal health, AD can be regarded as a fundamentally metabolic disease [10].

APP and Aβ, one of its cleavage products, have been shown to be relevant for mitochondrial dysfunction. Both have been found in the mitochondria of brain tissue in AD patients [11, 12]. At the molecular level, APP was reported to bind to the translocase of the outer mitochondrial membrane 40 (TOM40) as well as to the inner mitochondrial membrane import channel (TIM23) [13]. Forming stable complexes with the import channels, APP hinders transport of nuclear encoded proteins into the mitochondria, thereby affecting mitochondrial function [14]. Amyloid beta is thought to interact with several proteins amongst others some mitochondrial proteins, resulting in an increase in oxidative stress [15] which in turn could increase Aβ levels [16]. Regarding the mitochondrial respiratory system, *in vitro* studies on isolated mitochondria showed the reduced activity of pyruvate dehydrogenase and Cytochrome c Oxidase after Aβ treatment [17, 18]. Likewise, Aβ treatment of brain homogenate impaired mitochondrial oxygen consumption as well as mitochondrial membrane potential [19].

However, in intact cells, the mechanism of mitochondrial toxicity is more difficult to identify because the transport and localization of APP and its cleavage products play a major role [20]. Although mitochondrial alterations have been reported to be found mainly near amyloid plaques [21], there are controversial findings to the question of whether extracellular Aβ causes mitochondrial defects [22–24]. Interestingly, studies reporting a mitochondrial defect in a cell line or mouse model by overexpressing an AD-related protein mostly used APP or mutant forms of APP [25–29], resulting in both elevated levels of APP and Aβ. Thus, there is still a lack of clear evidence which protein is conferring the toxicity on mitochondria and how the localization might affect the toxicity.

To compare APP- versus Aβ mediated mitochondrial toxicity in intact cells we performed an overexpression of APP or BACE1 in human embryonic kidney cells (HEK293) and in a murine neuroblastoma cell line (N2a). Using high resolution respirometry, we demonstrate a reduction in mitochondrial respiration in APP-overexpressing cells, but no alterations upon BACE1 overexpression. Interestingly, the addition of a gamma secretase inhibitor could rescue the mitochondrial defect at a concentration not affecting the Aβ secretion rate but only intracellular Aβ levels. This unravels Aβ as the main toxic product and further highlights the necessity of its intracellular localization for affecting mitochondrial function.

## Materials and Methods

### Antibodies

For this study, the following primary antibodies were used: mouse (ms) anti-(α) APP N-terminus (22C11, Millipore/MAB348); rabbit (rb) α APP C-terminus (Sigma/A8717); ms α APP/Aβ (aa1-16, 6E10, Covance); ms α Aβ (aa17-24, 4G8, SIG-39200; Covance); rb α BACE1 (D10E5, Cell signaling); rb α hemagglutinin (HA) (Sigma/H6908); ms α c-myc (9E10, Sigma/M4439); ms α β-actin (AC-15, Sigma/A5441); and goat α ms antibody coupled to 10nM gold particles (Aurion, Wangeningen, The Netherlands). Secondary horseradish peroxidase coupled antibodies were obtained from Molecular Probes/Invitrogen.

### Expression constructs

HA-phCMV3 was purchased from Genlantis (product number: p003300). APP695 CMV(myc) is described in Kinoshita et al. [30]. BACE-phCMV3(HA) is described in von Einem et al. [31]. pUltra-hot is a lentiviral vector backbone for bi-cistronic expression of the gene of interest and the fluorescent reporter mCherry under the control of a human ubiquitin promoter. pUltra-hot was a gift from Malcolm Moore (Addgene plasmid # 24130) and served as control plasmid and as backbone for creation of APP-pUltra-hot and BACE pUltra-hot. BACE pUltra-hot was generated in our lab by subcloning BACE1 cDNA into the BamHI/XbaI cleavage sites of pUltra-hot. APP pUltra-hot was generated in our lab by subcloning APP695 cDNA into the XbaI/XmaI cleavage sites of pUltra-hot. psPAX2 is a packaging plasmid encoding for HIV-1 gag/pol sequences under the control of a SV40 promoter. psPAX2 was a gift from Didier Trono (Addgene plasmid # 12260). pMD2.G is an envelope expressing plasmid encoding for VSV-G glycoprotein under the control of a CMV promoter. pMD2.G was a gift from Didier Trono (Addgene plasmid # 12259).

### Lentiviral transduction system

The lentiviral expression vector pUltra-hot is deleted for all genes associated with packaging or replication of the virus. Just the information for bacterial replication, the terminal recombination sequences and the packaging signal is left. Thus, this 3^rd^ generation vector represents a very safe lentiviral system as the virus particles are not able to replicate. For the production of virus, LentiX 293T cells (Clonetech) were co-transfected (calcium phosphate transfection method) with pUltra-hot, psPax2 which encodes packaging proteins and pMD2.G which encodes for the envelope protein. Six hours after transfection, medium was changed to avoid transfection reagent in the conditioned medium to which the virus is secreted. Then, 48h after transfection conditioned medium was collected, filtered using a 0.2μM sterile filter (Sarstedt) and stored at -20°C.

### Cell culture and cell lines

The human embryonic kidney cell line HEK293 (DSMZ no.: ACC 305/ obtained 2008) and the murine neuroblastoma cell line N2a (DSMZ no.: ACC 148/ obtained 2013) were cultured in 75cm^2^ flasks under standard cell culture conditions in high glucose Dulbecco's Modified Eagle's Medium (Gibco) supplemented with 10% fetal calf serum (FCS) and 1% penicillin/streptomycin (P/S). HEK293 cells were transduced with pUltra-hot, APP pUltra-hot or BACE pUltra-hot using our lentiviral transduction system. Cells were cultivated for 14 days after transduction and were subsequently used in experimental procedures up to 14 days. Similar expression strength was controlled by FACS analysis of the fluorescent reporter (mCherry). Stable cell lines were created by the transfection of HEK293 cells (calcium phosphate transfection method) or N2a cells (SatisFection; Stratagene) with HA-phCMV3, APP695-CMV(myc) or BACE-phCMV3(HA). Then, 24h post transfection medium was supplemented with 400μg/ml neomycin for two weeks to achieve selection of transfected cells. Afterwards, neomycin concentration was reduced to 200μg/ml and after one week of adaption, cells were used for experiments. Cells treated with the gamma secretase inhibitor LY450139 (GSI) were cultivated in medium supplemented with GSI for 7 days prior to the first experiments. GSI concentration was kept stable by changing medium after 48h at the latest.

### High-resolution respirometry

Here, 48 h prior to respirometry cells were seeded in a 75cm^2^ flask (4.5million cells for HEK293, 1mio cells for N2a). Then, 18h prior to the measurement, medium was changed to synchronize the cells. At the day of measurement cells were trypsinized, counted and resuspended in their conditioned medium to a final concentration of 1mio/ml. Respirometry was performed in an Oxygraph-2k system (Oroboros Instruments, Innsbruck, Austria) calibrated to air (gain for oxygen sensor was set to 4) with standard cell culture medium. Cells were added to the two stirred (750rpm) chambers and chambers were sealed to obtain a closed system. Decreasing oxygen concentration in the chambers resembled cellular oxygen consumption. When oxygen consumption reached a plateau, a steady state level was obtained displaying Routine respiration. The addition of oligomycin (Olg) to a final concentration of 1.25μM resulted in Leak respiration. Subsequently, the proton gradient was released by stepwise titration (0.25μM FCCP/step) of the uncoupler FCCP (Carbonyl cyanide-*4*-(trifluoromethoxy)phenylhydrazone) until the maximum respiration was achieved (electron transport system capacity, ETS capacity). The addition of 0.5μM rotenone (Rot) and 5μM antimycin A (AA) blocked mitochondrial respiration completely, showing residual oxygen consumption (ROX). Subsequently, 2mM ascorbate (Asc) and 0.5μM TMPD were added, transferring electrons directly to complex IV. Due to auto-oxidation of ascorbate, the resulting oxygen consumption is dependent on the oxygen concentration in the chamber. By blockage of complex IV with 50μM sodium sulfide (Sulf), auto-oxidation and complex IV respiration could be separated. Analysis of the measurements was performed using DatLab version 5.1.0.20 (Oroboros Instruments, Innsbruck, Austria). Time intervals were drawn at the stable plateaus of oxygen flux quantifying the mean oxygen consumption of the respiratory states, which were corrected for ROX afterwards. For the calculation of complex IV respiration, three intervals before and six intervals after the injection of sodium sulfide were drawn at different oxygen concentrations. At intervals after the injection of sodium sulfide, the ratio of oxygen flux to oxygen concentration resembles the level of auto-oxidation of ascorbate. Subsequently, the portion of auto-oxidation-caused oxygen consumption in the first three intervals was calculated, thereby revealing complex IV capacity.

### Supernatant treatment

For collection of the supernatant HEK pUltra, HEK APP pUltra and HEK BACE pUltra were seeded like for high-resolution respirometry and after 30h medium was changes also according to respirometry experiments. After 48h this medium was removed and stored for a maximum of 10 days at 4°C before usage. Naïve HEK293 cells were treated with 10ml of this conditioned medium 18h before respirometry and also measured in this medium.

### Doubling time

Doubling time was determined in conjunction with the respirometry experiments. As the number of seeded cells was known and the cells were also counted before respirometry, this allowed calculation of the doubling time via the formula:

$$\text{Doubling time }\left(\text{h}\right)\;=\text{ culture time }\left(\text{h}\right)\;/{\text{ log}}_{2}\left({\text{cell number}}_{\text{end }}/{\text{ cell number}}_{\text{start}}\right)$$

### Lactic acid release

The samples for the glycolysis assay were also collected in conjunction with the respirometry experiments. Here, 1ml of the medium removed on the day of respirometry experiments was frozen at -20°C after centrifugation at 1000g for 5min. In those samples, the lactic acid concentration was determined using the glycolysis cell-based assay kit (Cayman chemicals), which was performed according to the manufacturer´s protocol with a 1:20 dilution of the samples. For the calculation of lactic acid release, the lactate concentration in fresh medium was subtracted from the lactate concentration in the samples. Subsequently, the lactic acid release was corrected for the collection time and the mean cell number during collection time.

### Transduction reduction assay

Transduction strength of HEK293 cells was quantified over a time period of 14 days using fluorescence activated cell sorting (FACS) analysis. Starting two weeks after transduction, cells were trypsinized every second or third day, washed once with Dulbecco´s Phosphate-buffered saline (DPBS) and resuspended in 200μl of DPBS. Triplicates were analyzed on a FACS Calibur (BD Biosicence) counting 20,000 cells per sample. The fluorescent reporter protein mCherry was detected in the FL3 channel and was quantified as the mean FL3 fluorescence. Fluorescence intensity for each time interval (5 days each, at least 2 time points of measurement per interval) was normalized to day 0 and subsequently normalized to control cells.

### Mitochondrial mass

Mitochondrial mass was measured using MitoTracker Green FM (Molecular Probes) and subsequent FACS analysis. For N2a, 1mio cells were seeded in a 75cm^2^ flask two days before the measurement. One day prior to the measurement, the medium was changed to synchronize the cells. At the day of measurement, cells were trypsinized, counted and for each condition 1mio cells were added to three FACS tubes. After washing cells with 1ml of DPBS, they were resuspended in 200μl OptiMEM supplemented with 50nM MitoTracker Green FM. Cells were stained for 30min at 37°C in the dark. After another washing step in DPBS, cell pellets were resuspended in 100μl 7-AAD staining solution (1:100 7-AAD in DPBS) for dead cell exclusion. After 10min of incubation at 37°C in the dark, cells were analyzed on a FACS Calibur (BD Bioscience) counting 20,000 cells per sample. HEK293 cells were seeded on a 12-well plate (3 wells per condition) at a density of 200,000 cells/well two days before the measurement. One day prior to the measurement, medium was changed to cell culture medium supplemented with 50nM MitoTracker Green FM and cells were stained overnight. Long-term incubation of HEK293 cells with MitoTracker Green FM resulted in better staining of the mitochondria, which was assessed by fluorescence microscopy. No reduced mitochondrial respiration could be detected after a 24h treatment of HEK293 cells with 50nM MitoTracker Green FM, indicating no mitochondrial toxicity conferred by 24h incubation with the dye. At the day of measurement, cells were trypsinized, transferred to FACS tubes, washed once with DPBS and subsequently analyzed on FACS. Samples were analyzed in triplicate and 20,000 cells were counted per sample. Dead cell exclusion for HEK293 cells was performed by gating in the forward and sideward scatter channel (FSC/SSC) and for N2a by excluding 7-AAD positive cells that were high in FL3 channel (620nm long pass filter). MitoTracker Green FM fluorescence was quantified using the mean intensity in the FL1 channel (525nm band pass filter). Additionally we determined citrate synthase activity as a marker for mitochondrial mass. For this purpose a commercially available kit was used according to manufacturer´s protocol (Sigma-Aldrich, CS0720). 40μg of total protein were applied for BEX cell lysates and 20μg for lysates in the provided lysis buffer of the kit. Only samples of the same lysis buffer and similar sample age (1 month range) were compared to avoid any bias in enzyme activity.

### Mitochondrial membrane potential

Mitochondrial membrane potential was evaluated using DiOC6, JC-1 and TMRM. HEK293 cells were seeded 2 days and synchronized by medium change 1 day prior to the measurement. For each condition, 1mio cells were added to six FACS tubes and washed with DPBS. JC-1 staining solution was prepared by mixing JC-1 stock (5mg/ml) with DMSO (1:3) and subsequent dilution in DMEM+10%FCS+1%P/S to a final concentration of 2.5μg/ml; cells were stained for 15min at 37°C in the dark. For staining cells with DiOC6 or TMRM, cells were incubated in DMEM+10%FCS+1%P/S supplemented with 25nM DiOC6 or 500nM TMRM, respectively, for 30min at 37°C in the dark. As a positive control for abolished membrane potential, 7.5μM of the uncoupler FCCP was added to the staining solution of three of the six tubes. Cells were analyzed on a FACS Calibur (BD Bioscience) counting 20,000 cells per sample. Dead cell exclusion was performed by gating in the FSC/SSC. Ratio of JC-1 monomers versus oligomers was detected by the ratio of mean green (FL1 channel) to mean orange/red (FL2 channel; 585 band pass filter) fluorescence. DiOC6 was detected as the mean FL1 channel intensity and TMRM as the mean FL2 channel intensity. Values were normalized to the mean of control cell triplicates and corrected for the uncoupled (FCCP-treated) control.

### Immunoelectron microscopy

Cells were seeded on pretreated sapphire disks [32] and cultivated for 48h before high-pressure freezing was performed according to Walther et al. [33]. Freeze-substitution was performed in medium consisting of acetone (VWR International GmbH, Darmstadt, Germany) with 0.1% uranyl acetate (Merck KGaA, Darmstadt, Germany) and 5% water [34]. Afterwards, cells were embedded in LR-Gold and cut according to Wilkat et al., 2014 [32]. For immunogold staining ultrathin sections were first incubated in 1% BSA for 10min and subsequently in ms α APP/Aβ-antibody (6E10) diluted 1:50 in 1% BSA blocking solution for 30min. After washing in blocking solution, sections were incubated in goat-anti mouse antibody coupled to 10nm gold particles (Aurion, Wageningen, The Netherlands) and post-fixed with glutaraldehyde and counter-stained with uranylacetate. Transmission electron microscopy (TEM) was performed using a JEM-1400 transmission electron microscope (Jeol GmbH, Eching, Germany) at an acceleration voltage of 120 kV. Images were recorded with a resolution of 2,048 × 2,048 pixel using a Veleta digital camera (Olympus Soft Imaging Solutions GmbH, Münster, Germany) and the iTEM software (Olympus Soft Imaging Solutions GmbH, Münster, Germany). At least 20 images of mitochondria-rich regions in different cells were acquired per condition. For analysis, the portions of mitochondrially-localized immunogold, corrected for the mitochondrial area in the respective image, were calculated. Thus the quantification is independent of absolute staining efficiency.

### sAPP and Aβ quantification

For determination of the secreted sAPP and Aβ in conditioned cell culture medium, 1ml of the medium was removed and stored at -20°C. For measurements of intracellular Aβ levels, cells were washed once with DPBS, harvested by 5min trypsinization, washed again with DPBS and finally 6mio cells were lysed in 200μl lysis buffer (Miltenyi Biotec). sAPP and Aβ levels were determined by sAPP Multiplex Assay Kit (K15120E), V-PLEX Aβ40 Peptide (4G8) Kit (K150SJE-1) or V-PLEX Aβ Peptide Panel 1 (4G8) Kit (K15199E-1), respectively, using the SECTOR Imager 2400 (Mesoscale Discovery), following the manufacturer’s instructions.

### Western blot analysis

Cell lysis was performed in BEX buffer (25mM Tris at pH 8.0, 20mM NaCl, 0.6% w/v deoxycholate and 0.6% Igepal CA-630). Gel electrophoresis was run under denaturing conditions with NuPage Novex Bis-Tris 4%-12% gradient gels (Invitrogen) and MOPS running buffer (Invitrogen). Semi-dry protein transfer on polyvinylidene difluoride membranes was followed by blocking in 1x RotiBlock (Roth) for 1 h. Incubation with primary antibodies was performed overnight at 4°C in 1x RotiBlock. Incubation with horseradish peroxidase-coupled secondary antibodies (1h, room temperature) and subsequent analysis with LAS-4000 (GE) was performed the following day.

### Statistical analysis

Statistical analysis was performed using GraphPad Prism 5 (GraphPad Software, Inc.). First, a D´Agostino & Pearson omnibus normality test was used to check for a Gaussian distribution of the data (significance level α = 0.05). For data in which all groups passed the normality test, an unpaired two-tailed t-test (for two groups) or a One Way ANOVA using Bonferroni’s pairwise multiple comparison (for more than two groups) was performed to check for significance. Non-normally distributed data were analyzed with a Mann-Whitney-U-test (two groups) or Kruskal-Wallis test and Dunn´s multiple comparison test (for more than two groups). Significance levels were defined as 0.05 (*), 0.01 (**) and 0.001 (***) and are indicated in the graphs.

## Results

### APP processing in HEK293 cells after overexpression of APP or BACE1

**Fig 1:**
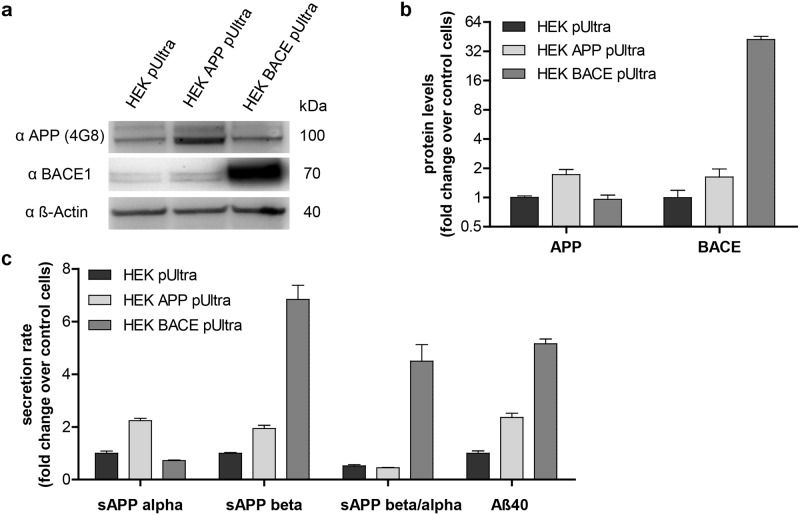
APP processing in HEK293 cells overexpressing APP or BACE1. **a)** APP and BACE1 expression in HEK293 cells transduced with APP pUltra-hot, BACE pUltra-hot or the empty control vector pUltra-hot detected by western blotting using 6E10 α-Aβ antibody (Covance) and D10E5 α-BACE1 antibody. β-actin as a loading control was detected using AC-15 α-β-actin antibody (Sigma). **b)** Fold change of protein expression in HEK APP pUltra and HEK BACE pUltra versus control cells (HEK pUltra) by quantification of western blots. Western blots of three independent protein samples were analyzed with a total of 9 images. APP and BACE1 levels were corrected for β-actin loading control and normalized to the levels of control cells, which were set to 1. Error bars indicate standard error. **c)** Quantification of sAPP and Aβ secretion rate using ELISA and corrected for the collection time and the mean cell number during collection time. Results were normalized to the mean values of control cells, which were set to 1. The mean values of 3 independent experiments measured in duplicates are shown. Error bars indicate standard error.

To investigate the effect of elevated levels of APP or Aβ on the mitochondria in intact cells, we performed a temporary stable overexpression of APP or BACE1 in HEK293 cells. As APP processing by BACE1 is the rate-limiting step in the amyloidogenic cleavage, low endogenous BACE1 expression in HEK293 cells makes them a good model for our study. Both proteins were overexpressed to a similar extent to avoid toxic effects of protein overexpression. This was assessed by quantification of the simultaneously expressed fluorescent reporter protein mCherry using FACS analysis. Overexpression resulted in a 2-fold increase of APP protein levels and a 40-fold increase in BACE1 levels (Fig 1a and 1b). To investigate alterations in the APP processing in our cells, we measured the levels of soluble amyloid precursor protein α or β (sAPP α/β) in conditioned media as markers of the non-amyloidogenic α-secretase cleavage or the Aβ-producing BACE1 cleavage, respectively. APP overexpression results in higher levels of sAPPα and sAPPβ but without changing their ratio compared to control cells (Fig 1c). However, BACE1 overexpression shifts APP cleavage towards the amyloidogenic pathway (Fig 1c). Consequently, determination of Aβ in the conditioned medium showed the highest Aβ secretion rate (5-fold increase) in HEK BACE pUltra and a 2-fold increase in HEK APP pUltra compared to control cells. To sum up, HEK APP pUltra display higher levels of APP as well as a modest increase in Aβ, whereas HEK BACE pUltra show a strong increase of Aβ secretion without alterations in APP protein levels.

### APP but not BACE overexpression results in reduced mitochondrial respiration in HEK293 cells

**Fig 2:**
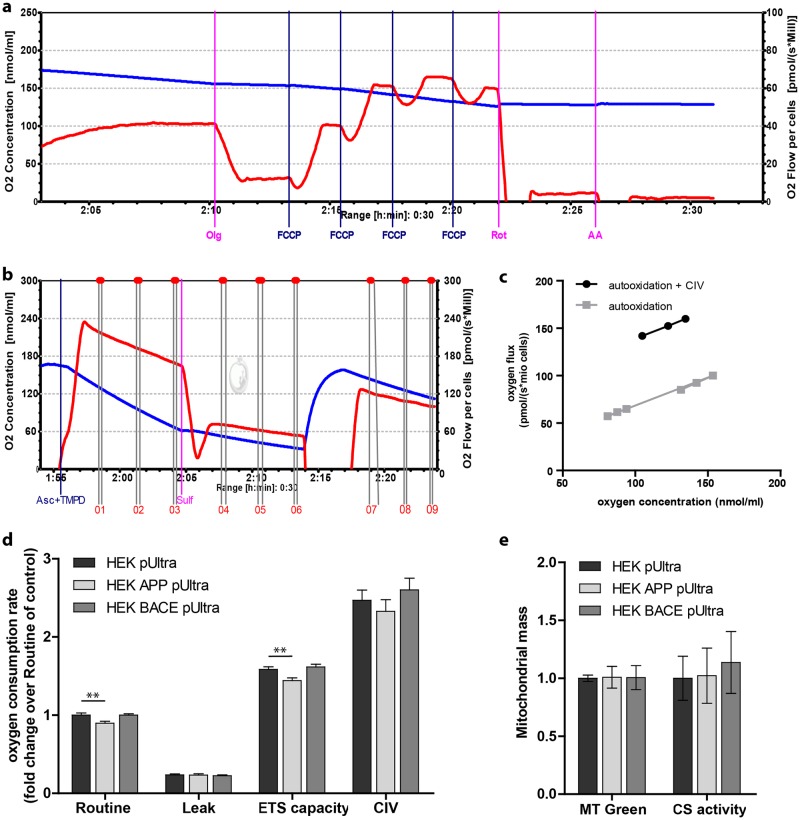
High-resolution respirometry reveals a reduced mitochondrial respiration following APP but not BACE overexpression. **a)** High-resolution respirometry performed in an Oroboros Oxygraph-2k. The blue curve shows the oxygen concentration in the sealed chamber and the red curve shows the oxygen consumption of the cells. Titration protocol: addition of 2mio cells in their conditioned medium (Routine respiration), 1.25μM oligomycin (Leak respiration), titration of FCCP to a final concentration of ~3μM (ETS capacity), 0.5μM rotenone and 5μM antimycin A (residual oxygen consumption; ROX). **b)** Measurement of complex IV capacity after blockage of complex III. Addition of 2mM ascorbate and 0.5mM TMPD (Asc+TMPD) results in maximum complex IV respiration plus auto-oxidation (intervals 1–3). Addition of 50μM sodium sulfide (Sulf) results in oxygen consumption just by auto-oxidation, which is dependent on oxygen concentration (intervals 4–9). **c)** Calculation of complex IV capacity. Pairs of values of oxygen flux and the corresponding oxygen concentration are displayed. The black rhombs show the dependence of the auto-oxidation on the oxygen concentration, whereas the blue squares show the sum of auto-oxidation and complex IV respiration in relation to the oxygen concentration. Thus, complex IV capacity can be seen as the distance between the blue and black regression curves. **d)** Oxygen consumption of the cells was corrected for ROX and normalized to the Routine respiration of HEK pUltra, which was set to 1. The mean of 7 (HEK pUltra), 8 (HEK APP pUltra) and 8 (HEK BACE) independent experiments, each measured in duplicate, are shown. Error bars indicate standard error. Significance versus control was calculated using Kruskal-Wallis test with Dunn´s method for correction of multiple comparison. **e)** Mitochondrial mass measured as Mitotracker Green FM fluorescence (MT Green) using FACS analysis or citrate synthase activity (CS activity). The mean values of 5 independent experiments measured in triplicate (MT Green) and 6 independent experiments measured in technical duplicates (CS activity) are shown, all normalized to control cells. Error bars indicate confidence intervals (95%). Significance versus control was calculated using Kruskal-Wallis test with Dunn´s method for correction of multiple comparison.

Next, we characterized mitochondrial function in HEK pUltra-hot, HEK APP pUltra and HEK BACE pUltra by performing high-resolution respirometry using an Oxygraph-2k (Oroboros Instruments). In order to investigate cellular respiration under physiological substrate supply, we chose to measure intact cells using an uncoupler inhibitor protocol (Fig 2a–2c). This revealed a significantly reduced routine respiration after APP but not BACE1 overexpression (Fig 2d). The addition of Oligomycin (Olg) resulted in Leak respiration displaying proton leak through the inner mitochondrial membrane, independently of complex IV. This respiratory state was not significantly altered between the cell lines (Fig 2d), indicating no alterations of the inner mitochondrial membrane integrity or proton associated transport processes. To investigate the functionality of the mitochondrial respiratory system independently of cellular energy demand, we added the uncoupler FCCP revealing the electron transport system capacity (ETS capacity). Similar to routine respiration, HEK APP pUltra showed a significant reduction in ETS capacity compared to control whereas ETS capacity in HEK BACE pUltra was not significantly altered (Fig 2d). Finally, the addition of rotenone (Rot) and antimycin A (AA) totally blocked respiration showing the residual oxygen consumption (ROX) which accounts for non-mitochondrial oxygen-consuming processes in the cells and which was corrected for in the analysis. As the results in the Oxygraph are displayed as respiration per cell number, alterations in mitochondrial content could account for differences in respiration. Therefore, we measured mitochondrial mass of the three cell lines by staining with Mitotracker Green FM (Molecular Probes) and subsequent FACS analysis and by analyzing citrate synthase activity. However, this revealed no significant alterations in mitochondrial content between the cell lines (Fig 2e). In order to exclude that mere transient stable expression of BACE1 results in the lack of altered mitochondrial respiration, we created HEK293 cells stably overexpressing BACE1 (HEK BACE-HA) under the control of a CMV promoter. However, similar to HEK BACE pUltra, mitochondrial respiration was not altered (S1 Appendix). To summarize, we could show that APP but not BACE1 overexpression in HEK293 cells reduces mitochondrial respiration and respiratory capacity.

### HEK APP pUltra display an increased mitochondrial membrane potential

**Fig 3:**
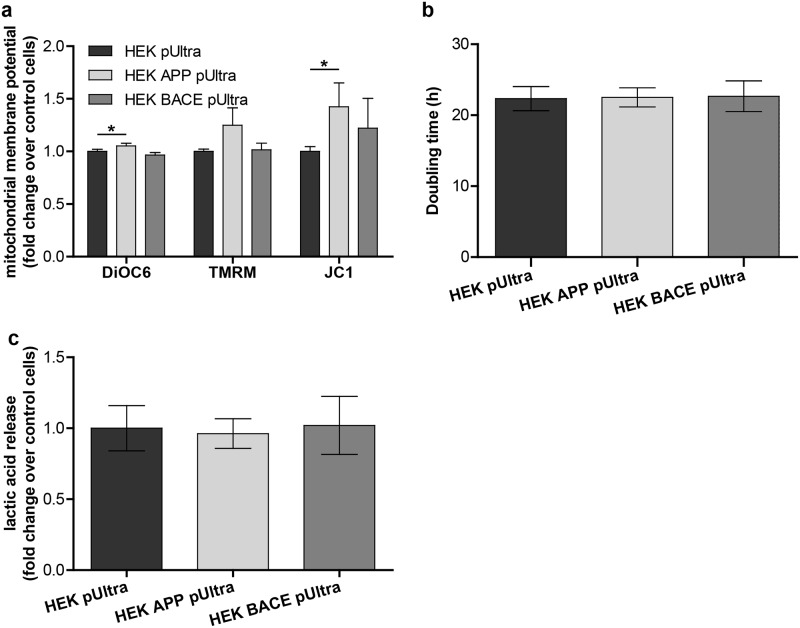
APP overexpression results in an increased mitochondrial membrane potential. **a)** Mitochondrial membrane potential was measured by staining the cells with the fluorescent dyes DiOC6, TMRM or JC-1 and subsequent analysis using FACS. Dead cells were excluded by gating in the FSC/SSC plot. Mean fluorescent intensities or the ratio of FL1/FL2 for JC-1 were corrected for the uncoupled control cells and normalized to control cells, which were set to 1. Means of 7 (DiOC6), 6 (TMRM) and 3 (JC-1) independent experiments are shown, each measured in triplicates. Error bars indicate standard error. Significance versus control was calculated using Kruskal-Wallis test with Dunn´s multiple comparison for DiOC6 and TMRM and One-way ANOVA with Bonferroni´s multiple comparison for JC-1. **b)** Calculated doubling time in the 48h period between seeding of the cells and high-resolution respirometry. The mean of 10 independent experiments is shown. Error bars indicate confidence intervals (95%). **c)** Measurement of lactic acid concentration in the conditioned medium in which also respirometry is performed in. Results of 5 independent experiments are shown, each measured in technical duplicates. Outcomes were corrected for collection time and mean cell number during collection time, and were finally normalized to the release rate of control cells, which was set to 1. Error bars indicate confidence intervals (95%).

As mitochondrial membrane potential (Δψ) is an important factor influencing ATP and ROS production, we determined Δψ in HEK pUltra, HEK APP pUltra and HEK BACE pUltra. We used three different dyes for measuring Δψ as they are considered controversial. Interestingly, all showed a tendency for a slight mitochondrial hyperpolarization in HEK APP pUltra, with DiOC6 and JC-1 reaching significance (Fig 3a). In contrast, no tendency was observed for HEK BACE pUltra (Fig 3a). Consequently, reduced routine respiration in HEK APP pUltra could also be a consequence of respiratory control, a mechanism that reduces electron transport system activity following mitochondrial hyperpolarization. Thus, to exclude the possibility of an intrinsic metabolic switch to a glycolytic phenotype in HEK APP pUltra, we analyzed cellular doubling time and lactic acid release. However, no significant alterations were found in the doubling time between the three cell lines (Fig 3b). Likewise, lactic acid release, estimating the glycolysis rate of the cells, was also not significantly altered (Fig 3c). This indicates the absence of an intrinsic origin of reduced routine respiration in HEK APP pUltra, strengthening the idea of a toxic effect. To sum up, HEK APP pUltra in contrast to HEK BACE pUltra displayed functional mitochondrial alterations.

### Stable murine neuroblastoma cells overexpressing APP exhibit reduced mitochondrial respiration

**Fig 4:**
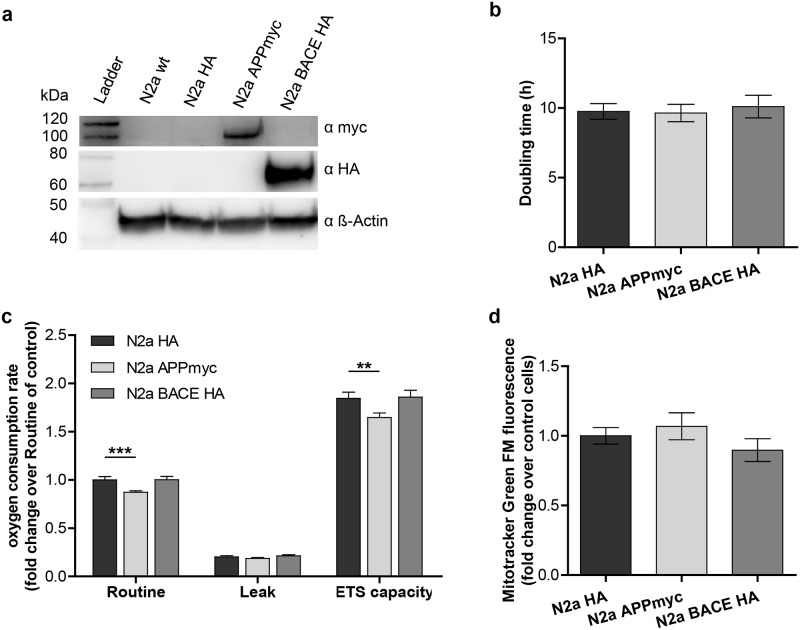
APP overexpression reduces mitochondrial respiration in Neuro-2a cells. **a)** APP and BACE1 overexpression in N2a cells stably transfected with HA-phCMV3, APP695-CMV(myc) or BACE-phCMV3(HA) detected by western blotting using α-HA antibody (Sigma) and 9E10 α-myc antibody (Sigma). β-actin as a loading control was detected using AC-15 α-β-actin antibody (Sigma). **b)** Calculated doubling time in the 48h period between seeding of the cells and high-resolution respirometry. The mean of 5 independent experiments is shown. Error bars indicate confidence intervals (95%). **c)** High-resolution respirometry of stably transfected N2a cells in an Oxygraph-2k. Titration protocol: addition of 2mio cells in their conditioned medium (Routine respiration), 1.25μM oligomycin (Leak respiration), titration of FCCP to a final concentration of ~2.5μM (ETS capacity), 0.5μM rotenone and 5μM antimycin A (ROX). Oxygen consumption of the cells was corrected for ROX and normalized to the Routine respiration of N2a HA, which was set to 1. The means of 10 independent experiments, each measured in duplicate, are shown. Error bars indicate standard error. Significance was calculated using Kruskal-Wallis test and Dunn´s multiple comparison test. **d)** Mitochondrial mass measured as Mitotracker Green FM fluorescence using FACS analysis. The mean fluorescence in the FL1 channel was normalized to control cells, which was set to 1. The mean values of 5 independent experiments measured in triplicate are shown. Error bars indicate confidence intervals (95%).

As HEK293 cells exhibit a high complex IV excess capacity (Fig 2d), we wanted to exclude cell line-specific characteristics of HEK293 cells resulting in the observed mitochondrial alterations. Thus, we created stable murine neuroblastoma cells overexpressing myc-tagged APP (N2a APPmyc), HA-tagged BACE1 (N2a BACE-HA) or HA-tag alone (N2a HA) (Fig 4a). Analyzing their doubling time revealed no significant alterations between the cell lines (Fig 4b). In contrast, high-resolution respirometry of intact cells using an Oxygraph-2k (Oroboros Instruments) showed a significant reduction in routine respiration as well as ETS capacity in N2a APPmyc, whereas no significant alterations were observed in N2a BACE-HA versus control (Fig 4c). No differences in the mitochondrial mass were detected that could account for reduced respiration following APP overexpression (Fig 4d). Together with the evidence in HEK293 cells, these results put forward a toxic effect of the amyloid precursor protein rather than of amyloid beta on the mitochondrial function.

### Treatment of HEK APP pUltra with a gamma secretase inhibitor rescues mitochondrial respiration

**Fig 5:**
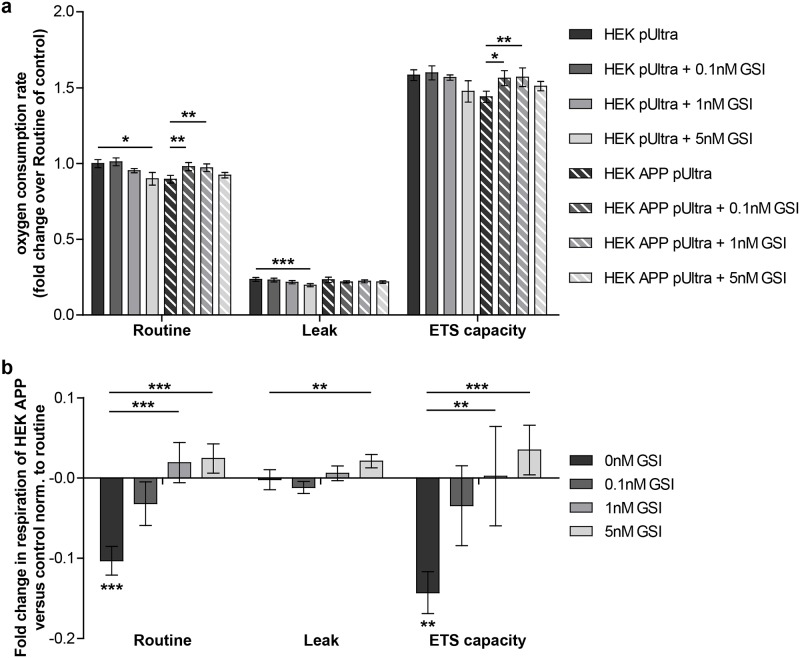
Gamma secretase inhibitor treatment rescues mitochondrial respiration in HEK APP pUltra. **a)** High-resolution respirometry of HEK pUltra and HEK APP treated with 0/0.1/1/5nM GSI, performed in an Oroboros Oxygraph-2k. Titration protocol: addition of 2mio cells in their conditioned medium (Routine respiration), 1.25μM oligomycin (Leak respiration), titration of FCCP to a final concentration of ~3μM (ETS capacity), 0.5μM rotenone and 5μM antimycin A (ROX). Oxygen consumption of the cells was corrected for ROX and normalized to the Routine respiration of HEK pUltra, which was set to 1. Means of at least 8 independent experiments measured in duplicates are shown. Error bars indicate standard error. Significance was evaluated for different GSI concentrations versus untreated control using Kruskal-Wallis test and Dunn´s multiple comparison test. **b)** Differences in respiration (normalized to Routine of control, which was set to 1) of HEK APP pUltra to the respective control cells treated with the same concentration of GSI. Lower asterisks indicate significances of HEK APP pUltra versus respective control, upper asterisks indicate significances between the differences (HEK APP pUltra to HEK pUltra) of GSI treated to untreated cells. Significance was calculated using Kruskal-Wallis test and Dunn´s multiple comparison test.

To check whether we can reverse the lower mitochondrial respiration by reducing Aβ production, we treated HEK pUltra and HEK APP pUltra with different concentrations of a gamma secretase inhibitor (GSI), reducing the final step of APP processing. We performed high-resolution respirometry using an Oxygraph 2-k (Oroboros Instruments). The treatment of control cells (HEK pUltra) with 0.1nM and 1nM GSI did not result in a significant difference of mitochondrial respiration at any respiratory state (Fig 5a). However a tendency towards a reduced respiration becomes visible at a concentration of 1nM becoming significant at 5nM GSI (Fig 5a). In contrast, the treatment of HEK APP pUltra with 0.1nM or 1nM GSI significantly improves absolute routine respiration as well as ETS capacity of those cells (Fig 5a). Normalizing the respiratory states to the respective control cells shows the effect of APP overexpression in the absence (black bars) and the presence (gray bars) of GSI (Fig 5b). This revealed a significant, stepwise reduction of the toxicity of APP overexpression on mitochondrial routine respiration and ETS capacity with increasing GSI concentrations (Fig 5b). These alterations are not due to differences in an altered doubling time (S2(a) Appendix) or in mitochondrial mass (S2(b) and S2(c) Appendix). Thus, these results indicate that a product of the final APP cleavage process confers the mitochondrial toxicity, most likely Aβ.

### Intracellular but not extracellular Aβ levels are associated with mitochondrial respiration

**Fig 6:**
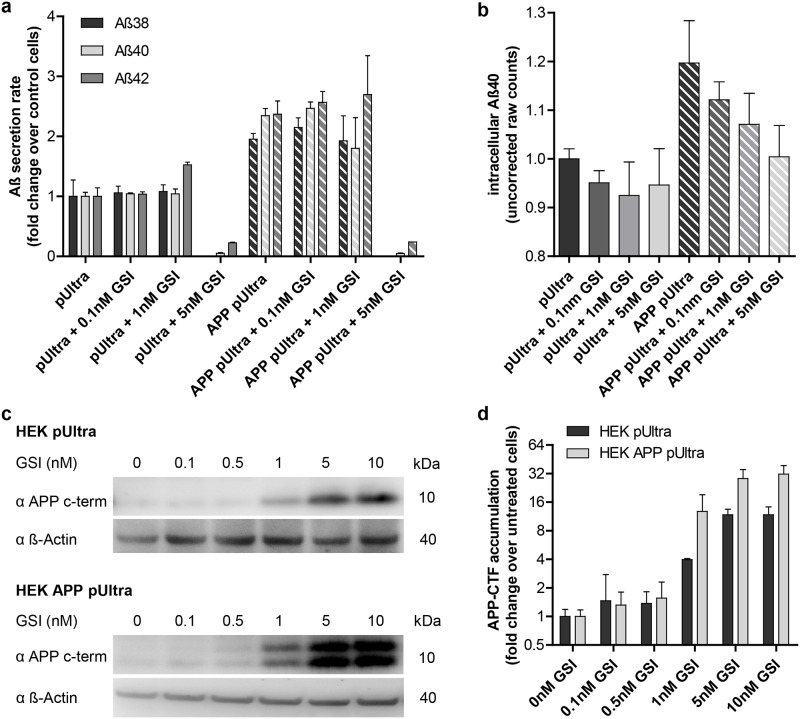
Intracellular but not extracellular Aß correlates with the rescue of mitochondrial respiration following GSI treatment. **a)** Quantification of Aβ38, Aβ40 and Aβ42 secretion rate using ELISA. Secretion rates were corrected for the collection time and the mean cell number during collection time. Results were normalized to the mean values of untreated control cells, which were set to 1. The mean values of at least 3 independent experiments measured in technical duplicates are shown. Error bars indicate standard error. **b)** Intracellular Aβ40 levels measured by ELISA and displayed as absolute raw counts normalized to untreated control cells, which were set to 1. Error bars indicate standard error. **c)** Accumulation of APP-CTF detected by western blotting using α APP C-terminus-antibody (Sigma). **d)** Fold change of APP-CTF accumulation in HEK pUltra and HEK APP pUltra normalized to untreated cells (set to 1) by quantification of western blots. Western blots of three independent protein samples per condition were analyzed with 3 images each. Equal loading was ensured by BCA protein assay. Error bars indicate standard error.

Interestingly, measuring the levels of secreted Aβ in the conditioned medium of HEK pUltra or HEK APP pUltra revealed no significant reduction of Aβ38, Aβ40 or Aβ42 at concentrations of 0.1nM or 1nM GSI (Fig 6a). Only treatment with 5nM GSI reduced extracellular Aβ levels to less than 20% of respective control (Fig 6a). In contrast, analyzing the effect of GSI treatment on the APP c-terminal fragments reveals a significant accumulation of C99 and C83 already at a lower concentration (Fig 6c and 6d). Thus, we observed an accumulation of APP c-terminal fragments preceding the reduction of extracellular Aβ. As an accumulation of C83 goes along with the reduced production of Aβ, we hypothesized that the intracellular Aβ pool is reduced first after GSI treatment. Consequently, we measured Aβ in cell lysates of HEK pUltra and HEK APP treated with different concentrations of GSI via ELISA. Uncorrected raw counts of Aβ40 show a clear tendency of reduced intracellular Aβ40 already at a concentration of 0.1nM GSI (Fig 6b). This indicates an association of GSI-mediated rescue of mitochondrial respiration with intracellular Aβ levels (Figs 5b and 6b).

### APP but not BACE1 overexpression in HEK293 cells raises intracellular Aβ

**Fig 7:**
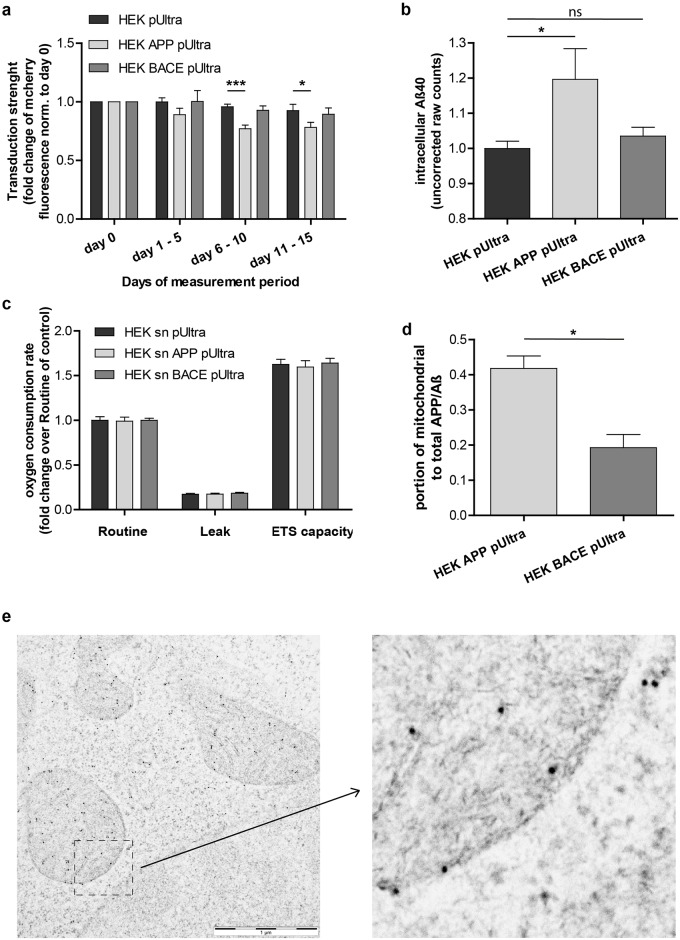
APP as against BACE1 overexpression results in higher intracellular Aβ and a reallocation towards mitochondria. **a)** Reduction of transduction strength quantified by measuring mCherry fluorescence of living cells by FACS analysis. Fluorescence was normalized to the first day of measurement (day 0) which was set to 1. Means of 3 independent experiments each with at least 2 time points of analysis per time interval are displayed. Error bars indicate standard error. Significance versus control cells was calculated using One-way ANOVA with Bonferroni´s Multiple comparison. **b)** Intracellular Aβ40 levels measured by ELISA and displayed as absolute raw counts normalized to control cells, which were set to 1. Error bars indicate standard error. Significance was calculated using unpaired two-tailed t-test. **c)** High-resolution respirometry of naïve HEK293 cells treated with conditioned medium from HEK pUltra, HEK APP pUltra or HEK BACE pUltra for 18h, Oxygen consumption of the cells was corrected for ROX and normalized to the Routine respiration of HEK sn pUltra, which was set to 1. Means of 6 independent experiments measured in duplicates are shown. Error bars indicate standard error. Significance was evaluated versus control using Kruskal-Wallis test with Dunn´s multiple comparison for routine respiration and One-way ANOVA with Bonferroni´s multiple comparison for Leak respiration and ETS capacity. **d)** Quantification of mitochondrially localized portion of APP/Aβ determined by immunoelectron microscopy. Per experiment, at least 20 images of mitochondria-rich regions were taken and all immunogold particles were counted manually. Means of 3 independent experiments are displayed. Error bars indicate standard error. Significance was calculated using unpaired two-tailed t-test. **e)** Exemplary image for the immunogold-staining of Aβ/APP in HEK293 cells.

Consequently, we questioned whether intracellular Aβ levels could be the reason for having a reduced mitochondrial respiration after APP but not after BACE1 overexpression. First evidence of an intracellularly localized toxic product is provided by our “transduction reduction assay”. Using FACS analysis, we monitored expression strength as the fluorescence intensity of the reporter fluorophore mCherry. As the constructs stably integrate into the genome of the cells, reduction of expression strength results from selective advantage of untransduced cells over transduced cells. Both populations are present in the same culture dish, and are therefore exposed to the same extracellular components. Consequently, after correcting for protein-overexpressing stress by normalization to the control cells (HEK pUltra-hot), the significantly faster transduction reduction in HEK APP pUltra (Fig 7a) indicates an intracellular toxin rather than an extracellular one. In contrast, HEK BACE pUltra does not show a faster transduction reduction compared to control (Fig 7a). Measuring intracellular Aβ-levels in HEK pUltra-hot, HEK APP pUltra and HEK BACE-pUltra revealed significantly higher intracellular Aβ levels in APP but not BACE1 overexpressing cells compared to control cells (Fig 7b), which further strengthens the evidence of intracellular Aβ to be the toxic product. Finally, to crosscheck that extracellular Aβ really does not convey any effect on mitochondrial respiration we treated naïve HEK293 cells with conditioned medium of HEK pUltra (HEK sn pUltra), HEK APP (HEK sn APP) or HEK BACE (sn BACE) for 18h before analyzing them by high-resolution respirometry. We could show that treatment with supernatant of HEK APP or HEK BACE cells does not have any effects on mitochondrial respiration of naïve HEK293 cells (Fig 7c). This puts forward that the intracellular Aβ confers the toxic effects on mitochondria. Interestingly intracellular Aβ levels are opposite to Aβ secretion rates comparing HEK APP pUltra and HEK BACE pUltra(see Fig 1c). This indicates alterations in the localization of APP processing and subsequent Aβ transport. Analyzing intracellular APP/Aβ localization using immunoelectron microscopy showed that APP compared to BACE overexpression in HEK293 cells results in a higher portion of mitochondrial APP/Aβ (Fig 7d). Thus, this suggests that this reallocation of APP/Aβ to the mitochondria could be a determining factor in Aβ disturbing mitochondrial function. Furthermore, it indicates that Aβ toxicity on mitochondria is rather a direct effect than mediated by signaling cascades. To conclude, these findings highlight the prominent role of intracellular Aβ and its subcellular localization, especially in the mitochondria, in AD-associated mitochondrial dysfunction.

## Discussion

Bioenergetic deficits are widely accepted as an important factor in the pathophysiology of Alzheimer´s disease [35] with mitochondria being the main source of ATP in high energy-consuming neurons [36]. Aβ, as well as APP, have been described to exert manifold actions on mitochondria [37, 38], finally impairing mitochondrial respiration. However, in intact cells, it is unclear to which product of APP processing the reduced mitochondrial respiration has to be attributed to, with APP or Aβ being the main candidates. Therefore, we addressed this question in the present study by elevating APP and Aβ levels or Aβ levels alone in two distinct cell lines by the overexpression of APP or BACE1, respectively. Similar to other studies [25, 27, 28, 39] we observed a reduced mitochondrial respiration in cell lines overexpressing APP although the magnitude of reduction in our cells was lower. This could be due to the moderate increase in APP expression levels or due to the fact that we performed respirometry of intact instead of permeabilized cells. However, we did not observe a decrease in mitochondrial membrane potential but rather an increase. Upon first glance, this might be opposed to the reduced respiration. Indeed, this coincidence could be due to respiratory control, which intrinsically reduces respiration during mitochondrial hyperpolarization to prevent excessive ROS production [40–42]. Alternatively, it was shown that CI inhibition in HEK293 cells results in a hyperpolarization of mitochondrial membrane potential [43]. Thus, it might be cell-specific characteristics, indicating a defect in complex I respiration in APP-overexpressing cells. In contrast, previous studies mainly reported a deficiency of the Cytochrome c Oxidase [44, 45]. We detected a non-significant decrease in complex IV respiration, maybe failing to reach significance due to the complex calculation procedure and an accompanying high standard deviation. In fact, the uncoupler-inhibitor protocol chosen for respirometry does not allow for a more detailed analysis of the complexes involved in the mitochondrial defect but rather provides a near-physiological measurement of oxygen consumption with endogenous substrate [46].

Although the lack of mitochondrial dysfunction in BACE-overexpressing cells indicated APP-mediated toxicity, rescue of mitochondrial respiration by the addition of a gamma secretase inhibitor suggested Aβ as the toxic product. This is in line with other reports describing a beneficial effect of gamma secretase inhibitors and modulators on mitochondrial function in AD model systems [47, 48]. However, as we observed a rescue effect of GSI at concentrations not affecting Aβ secretion, other side effects of GSI have to be considered. In fact, GSI also results in an accumulation of the APP c-terminal fragments, a reduction in AICD release and an inhibition of the processing of other gamma secretase substrates like Notch. Downregulation of the APP intracellular domain (AICD) or Notch signaling by addition of a GSI was shown to rather reduce mitochondrial oxygen consumption or activity of the respiratory complexes [49] [50]. Concerning the increased levels of C99 and C83, to our knowledge, there are no studies reporting a beneficial effect on mitochondrial respiration. In contrast, C99 is rather ascribed to impair mitochondria by either a direct interaction [51] or by an altered mitochondrial trafficking in neurites [52]. Thus, these side effects of GSI treatment are not likely to account for the rescue of mitochondrial respiration in HEK APP cells but rather explain the reduced respiration in control cells treated with the highest concentration of GSI. Interestingly, similar to our observation, Mitani et al. [53] observed the accumulation of C99 preceding the reduction of Aβ secretion following treatment of cells with the gamma secretase inhibitor LY450139. This indicates another pool of Aβ to be reduced, which is in line with a trend towards the reduction of intracellular Aβ40. Thus, our results further point to a more prominent role of intracellular versus extracellular Aβ in mitochondrial dysfunction and a minor role of Aβ uptake in those cells. However, this might be cell-specific, as Cha et al. [24] reported mitochondrial alterations after the endocytosis of extracellular Aβ42. Indeed, the idea of intracellular Aβ toxicity has strengthened in recent years [54–57]. In particular, Aβ accumulation in mitochondria has been reported in several studies [58] potentially deriving from an Aβ release in mitochondria-associated membranes [59] or even in mitochondria [60]. In this connection the lack of mitochondrial dysfunction in BACE1 overexpressing cells, showing the highest Aβ secretion rate, is not a surprise. However, high overexpression of BACE1, as present in our cells, was reported to alter subcellular localization of APP cleavage and subsequent Aβ deposition [61]. Likewise, in BACE1-overexpressing cells, we observed a reduced percentage of APP/Aβ localized in the mitochondria and no increased levels of intracellular Aβ compared to control cells. Thus, we hypothesize that the altered localization of APP cleavage in BACE1 overexpressing HEK293 and N2a cells results in less mitochondrial Aβ. This idea is further underlined by the reported associations of mitochondrial Aβ and mitochondrial dysfunction [62].

To sum up, our results suggest that the mitochondrial respiration in intact cells is mainly affected by increased levels of Aβ rather than APP. In addition, we report a prominent role of intracellular in contrast to extracellular Aβ for mitochondrial toxicity. This highlights the importance of studying intact systems as transport processes and subsequent localization of APP and its cleavage products are a determining factor for their toxicity.

## Conclusion

Comparing the effects of the amyloid precursor protein and amyloid beta on mitochondrial function in intact cell lines, we found clear evidence for amyloid beta as the main toxic product resulting in lower mitochondrial respiration. In addition, our study revealed the importance of an intracellular, presumably mitochondrial, localization of Aβ for conferring a mitochondrial toxicity. This underlines the subcellular localization of APP processing and Aβ release as a decisive factor in the development of mitochondrial dysfunction. Especially for highly branched neurons, in which transport processes are even more important, investigating and modifying Aβ localization and the accompanying region-specific mitochondrial defects might be a promising approach for the future.

## Supporting Information

S1 Appendix Mitochondrial respiration in HEK293 stably overexpressing BACE1 is not altered. **a)** BACE expression in HEK293 cells stably transfected with BACE-phCMV3(HA) or the empty control vector HA-phCMV3 detected by western blotting using D10E5 α-BACE antibody. β-actin as a loading control was detected using AC-15 α-β-actin antibody (Sigma). **b)** Fold change of sAPP and Aβ40 secretion rates measured using ELISA. Results were corrected for the collection time and the mean cell number during collection time and normalized to the mean values of control cells, which were set to 1. The mean values of 3 independent experiments measured in duplicate are shown. Error bars indicate standard error. **c)** High-resolution respirometry performed in an Oroboros Oxygraph-2k. Titration protocol: addition of 2mio cells in their conditioned medium (Routine respiration), 1.25μM oligomycin (Leak respiration), titration of FCCP to a final concentration of ~3μM (ETS capacity), 0.5μM rotenone and 5μM antimycin A (residual oxygen consumption; ROX). Oxygen consumption of the cells was corrected for ROX and normalized to the Routine respiration of control cells, which was set to 1. Means of at least 7 independent experiments measured in duplicates are shown. Error bars indicate standard error. Significance versus control was evaluated using an unpaired two-tailed t-test. **d)** Mitochondrial mass measured as Mitotracker Green FM fluorescence using FACS analysis. The mean fluorescence in the FL1 channel was normalized to control cells, which was set to 1. The mean values of 5 independent experiments measured in triplicates are shown. Error bars indicate confidence intervals (95%).
(TIF)

S2 Appendix Doubling time and mitochondrial mass in HEK293 cells treated with GSI. **a)** Calculated doubling time in the 48h period between seeding of the cells and high-resolution respirometry. The mean of at least 8 independent experiments is shown. Error bars indicate confidence intervals (95%). **b)** Mitochondrial mass measured as Mitotracker Green FM fluorescence using FACS analysis. The mean fluorescence in the FL1 channel was normalized to untreated control cells, which was set to 1. The mean values of 5 independent experiments measured in triplicates are shown. Error bars indicate confidence intervals (95%). c) Mitochondrial mass measured as citrate synthase activity (CS activity). The mean values of 3 independent experiments measured in technical duplicates are shown, all normalized to control cells. Error bars indicate standard error.
(TIF)
