# Supplementary figures and images for: Metabolic Characterization of Intact Cells Reveals Intracellular Amyloid Beta but Not Its Precursor Protein to Reduce Mitochondrial Respiration
Source: PLoS One. 2016 Dec 22;11(12):e0168157. doi: 10.1371/journal.pone.0168157 (PMC5178995; doi:10.1371/journal.pone.0168157)

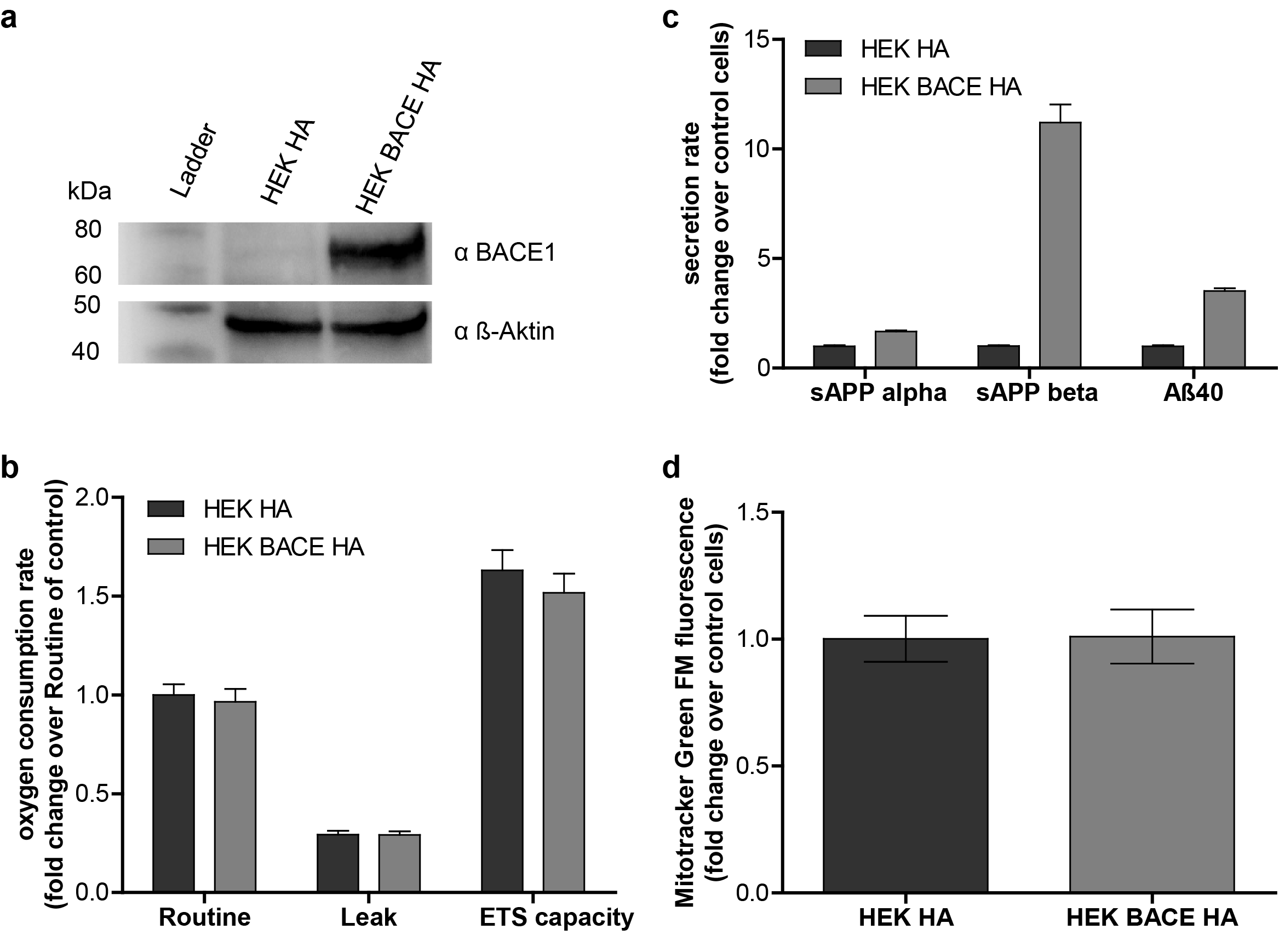

Supplement: S1 Appendix — a) BACE expression in HEK293 cells stably transfected with BACE-phCMV3(HA) or the empty control vector HA-phCMV3 detected by western blotting using D10E5 α-BACE antibody. β-actin as a loading control was detected using AC-15 α-β-actin antibody (Sigma). b) Fold change of sAPP and Aβ40 secretion rates measured using ELISA. Results were corrected for the collection time and the mean cell number during collection time and normalized to the mean values of control cells, which were set to 1. The mean values of 3 independent experiments measured in duplicate are shown. Error bars indicate standard error. c) High-resolution respirometry performed in an Oroboros Oxygraph-2k. Titration protocol: addition of 2mio cells in their conditioned medium (Routine respiration), 1.25μM oligomycin (Leak respiration), titration of FCCP to a final concentration of ~3μM (ETS capacity), 0.5μM rotenone and 5μM antimycin A (residual oxygen consumption; ROX). Oxygen consumption of the cells was corrected for ROX and normalized to the Routine respiration of control cells, which was set to 1. Means of at least 7 independent experiments measured in duplicates are shown. Error bars indicate standard error. Significance versus control was evaluated using an unpaired two-tailed t-test. d) Mitochondrial mass measured as Mitotracker Green FM fluorescence using FACS analysis. The mean fluorescence in the FL1 channel was normalized to control cells, which was set to 1. The mean values of 5 independent experiments measured in triplicates are shown. Error bars indicate confidence intervals (95%). (TIF) [file pone.0168157.s001.tif]

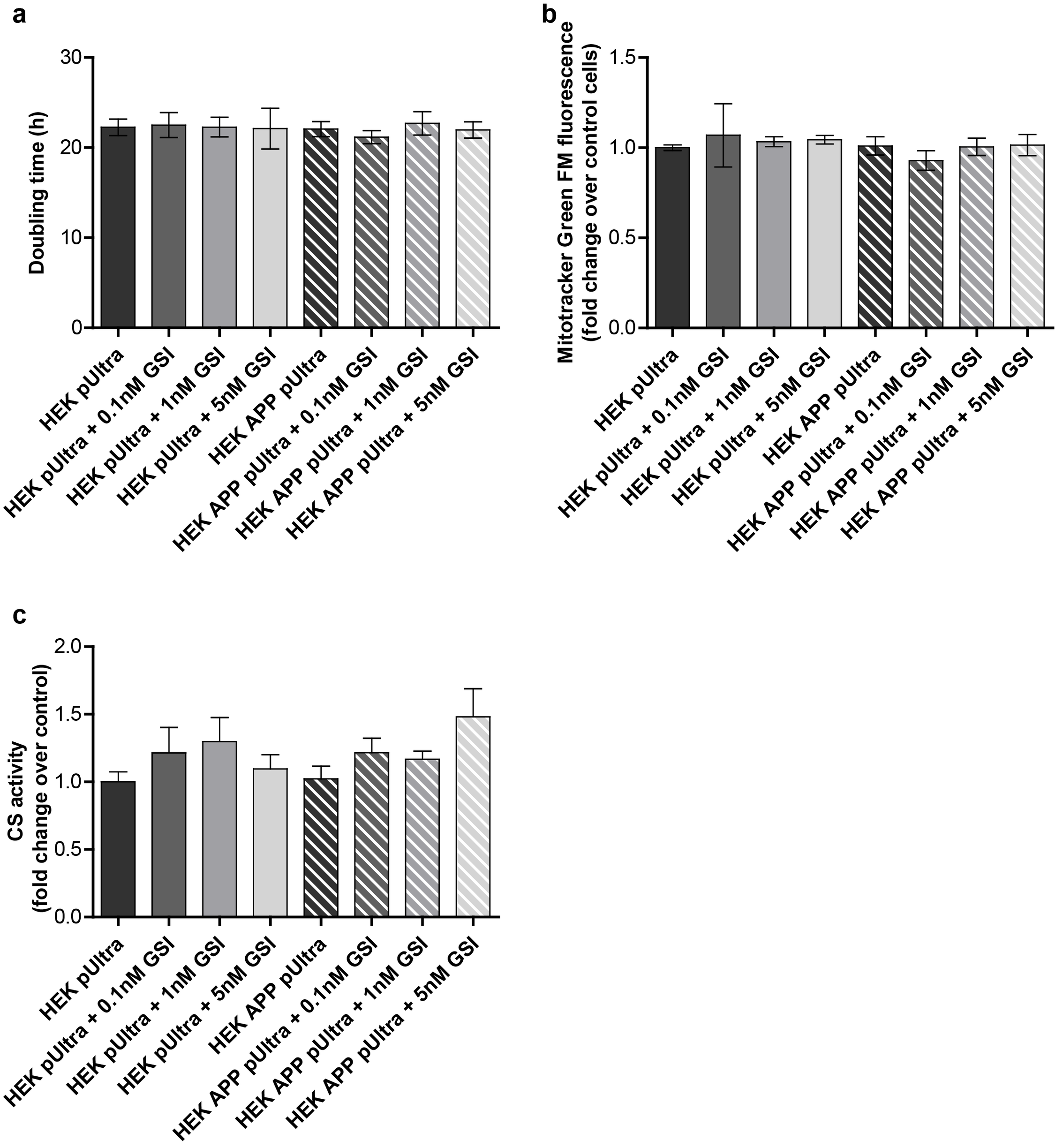

Supplement: S2 Appendix — a) Calculated doubling time in the 48h period between seeding of the cells and high-resolution respirometry. The mean of at least 8 independent experiments is shown. Error bars indicate confidence intervals (95%). b) Mitochondrial mass measured as Mitotracker Green FM fluorescence using FACS analysis. The mean fluorescence in the FL1 channel was normalized to untreated control cells, which was set to 1. The mean values of 5 independent experiments measured in triplicates are shown. Error bars indicate confidence intervals (95%). c) Mitochondrial mass measured as citrate synthase activity (CS activity). The mean values of 3 independent experiments measured in technical duplicates are shown, all normalized to control cells. Error bars indicate standard error. (TIF) [file pone.0168157.s002.tif]
